# Understanding the Hsp90 *N*-Terminal Dynamics: Structural and Molecular Insights into the Therapeutic Activities of Anticancer Inhibitors Radicicol (RD) and Radicicol Derivative (NVP-YUA922)

**DOI:** 10.3390/molecules25081785

**Published:** 2020-04-13

**Authors:** Ayanda M. Magwenyane, Ndumiso N. Mhlongo, Monsurat M. Lawal, Daniel G. Amoako, Anou M. Somboro, Sphelele C. Sosibo, Letitia Shunmugam, Rene B. Khan, Hezekiel M. Kumalo

**Affiliations:** 1 Drug Research and Innovation Unit, Discipline of Medical Biochemistry, School of Laboratory Medicine and Medical Science, University of KwaZulu-Natal, Durban 4000, South Africa; 209512288@stu.ukzn.ac.za (A.M.M.); Mhlongon4@ukzn.ac.za (N.N.M.); lawalmonsurat635@gmail.com (M.M.L.); dasticky2010@gmail.com (D.G.A.); anou.somboro@gmail.com (A.M.S.); Letitias15@gmail.com (L.S.); myburgr@ukzn.ac.za (R.B.K.); 2 Biomedical Resource Unit, College of Health Sciences, University of KwaZulu-Natal, Durban 4000, South Africa; 3 School of Physical and Chemical Sciences, Department of Chemistry, North West University, Mafikeng Campus, Mmabatho 2790, South Africa; sphegema@yahoo.com

**Keywords:** Hsp90 protease, *N*-terminal, radicicol, NVP-AUY922, density functional theory (DFT), molecular dynamics (MD), MM/GBSA

## Abstract

Heat shock protein 90 (Hsp90) is a crucial component in carcinogenesis and serves as a molecular chaperone that facilitates protein maturation whilst protecting cells against temperature-induced stress. The function of Hsp90 is highly dependent on adenosine triphosphate (ATP) binding to the *N*-terminal domain of the protein. Thus, inhibition through displacement of ATP by means of competitive binding with a suitable organic molecule is considered an attractive topic in cancer research. Radicicol (RD) and its derivative, resorcinylic isoxazole amine NVP-AUY922 (NVP), have shown promising pharmacodynamics against Hsp90 activity. To date, the underlying binding mechanism of RD and NVP has not yet been investigated. In this study, we provide a comprehensive understanding of the binding mechanism of RD and NVP, from an atomistic perspective. Density functional theory (DFT) calculations enabled the analyses of the compounds’ electronic properties and results obtained proved to be significant in which NVP was predicted to be more favorable with solvation free energy value of −23.3 kcal/mol and highest stability energy of 75.5 kcal/mol for a major atomic delocalization. Molecular dynamic (MD) analysis revealed NVP bound to Hsp90 (NT-NVP) is more stable in comparison to RD (NT-RD). The Hsp90 protein exhibited a greater binding affinity for NT-NVP (−49.4 ± 3.9 kcal/mol) relative to NT-RD (−28.9 ± 4.5 kcal/mol). The key residues influential in this interaction are Gly 97, Asp 93 and Thr 184. These findings provide valuable insights into the Hsp90 dynamics and will serve as a guide for the design of potent novel inhibitors for cancer treatment.

## 1. Introduction

Cancer is one of the world’s leading causes of death, responsible for approximately 9.8 million deaths in 2018 [1]. The commonly occurring malignancies include: lung, liver, colorectal, gastrointestinal and breast cancer [1]. The incidence rates of cancer are majorly influenced by increased exposure to several risk factors such as tobacco use, obesity, sedentary lifestyles, alcohol consumption, sun exposure, urbanization as well as environmental and occupational exposures [2]. The onset of carcinogenesis can also be facilitated by hereditary genetic mutations and auto-immune disorders, abnormal metabolic processes or as a secondary illness during hepatitis and human papillomavirus (HPV) infections [3]. At present, several targeted novel strategies are utilized in cancer therapeutics, which directly affect the cellular machinery involved in modulation of growth signal transduction, cell invasion, metastatic spread, apoptosis, cell cycle and tumor-related angiogenesis [4]. Hence, inhibition of the mentioned stages has become an attractive target in cancer treatment development. Furthermore, the approach to inhibiting tumor energy metabolism has proven a promising strategy [5].

The current cancer therapies available are limited and are usually in the form of tumorigenic removal, chemotherapy and radiotherapy. These treatments are often regarded as harmful and invasive, and the success rate is highly variable amongst patients. Over the years, chemotherapy has remained a popular route in the fight against cancer; however, this method is abrasive and debilitating to patients undergoing treatment due to the harsh chemical cocktails used. Unfortunately, many patients succumb to the effects of cancer due to unsuccessful therapeutic intervention caused by aggressive malignancies or relapsed remission. For this reason, continuous research is imperative for the design and discovery of novel chemotherapeutic drugs that can be used as potent anti-cancer agents. 

**Figure 1:**
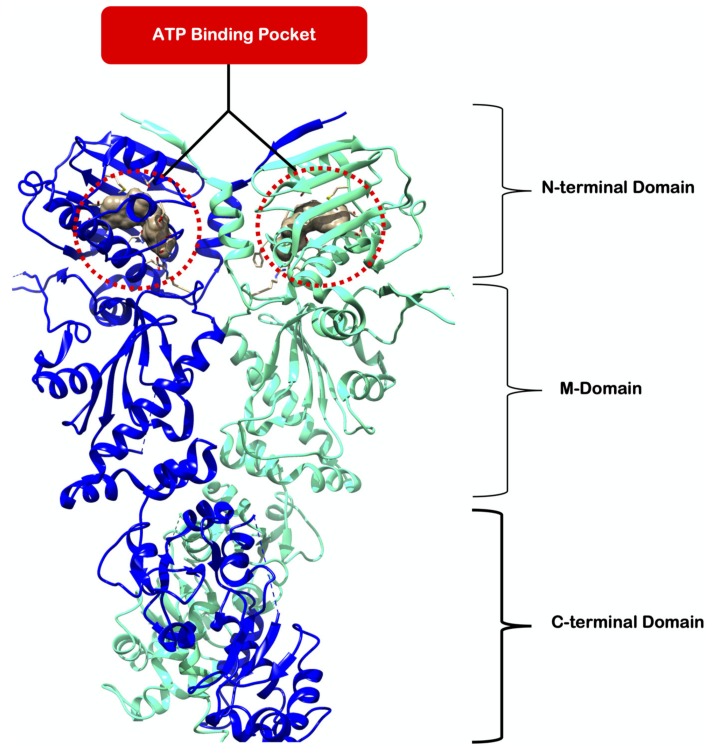
Crystal structure of Hsp90 dimer (PDB ID: 2CG9) [13]. The red dashed cycle highlights an ATP-binding pocket.

Heat shock protein 90 (Hsp 90, Figure 1) is a molecular chaperone that facilitates protein maturation, folding and assembly whilst minimizing the danger of aggregation within the protein intercellular environment. The Hsp90 protein is crucial for the stability and function of several cancer-related proteins, such as anaplastic lymphoma kinase (ALK), v-raf murine sarcoma viral oncogene homolog B1 (BRAF), epidermal growth factor receptor (EGFR), ErbB family 2 (ERBB2), insulin-like growth factor-1 receptor (IGF1R), v-kit Hardy-Zuckerman 4 feline sarcoma viral oncogene homology (KIT), and Met proto-oncogene [6]. As a result, scientists from the medicinal chemistry and pharmaceutical industry have placed great effort into Hsp90-cancer research to establish an effective strategy against cancer development and progression [7,8,9,10,11,12]. 

**Figure 2:**
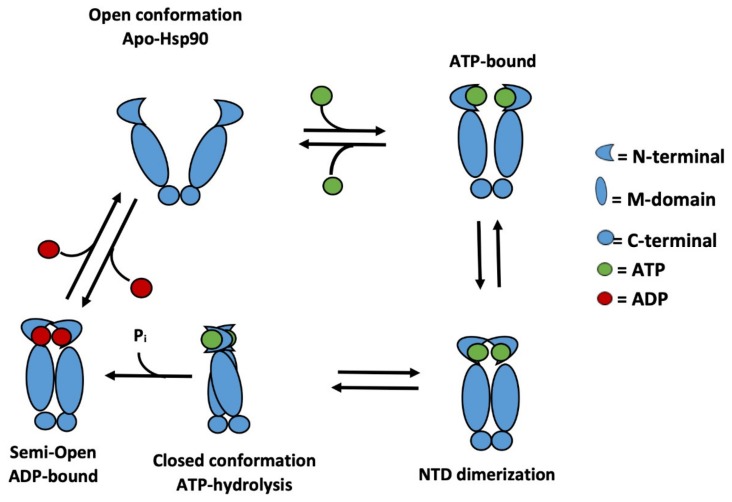
Schematic representation of the Hsp90 ATPase cycle [14].

The Hsp90 protein consists of three domains: (1) *N*-terminal, (2) middle domain and (3) C-terminal domain (Figure 1). Each domain has a functional role in the activity of Hsp90. The *N*-terminal domain contains the ATP-binding pocket. The protein catalyzes ATP decomposition to generate adenosine diphosphate (ADP) and a free phosphate ion, thus appropriately adopting the term “ATPase”. Signaling proteins, also referred to as client proteins, bind to the C-terminal of ATPase where they undergo maturation and folding. The schematic representation of the Hsp90 ATPase cycle mechanism is presented in Figure 2. Initially, Hsp90 homodimer adopts an open V-shaped conformation. Once ATP binds to the active site, dimerization of the *N*-terminal domains of each homodimer is prompted. This action is followed by the subsequent closure of Hsp90 and the M-domain is recruited for ATP hydrolysis. The ADP-bound conformation renders the dimers into a semi-open intermediate conformational state. The ADP is release and the ATPase cycle is ready to restart [14].

Radicicol (RD) and geldanamycin (GA) have been identified as natural inhibitors, binding directly to ATP-pocket in the *N*-terminal domain, thus competing with ATP. In 1953, Delmotte and Delmotte-Plaquee [15] discovered RD through isolation from monosporium bonorden and used the compound as a macrocyclic lactone antibiotic. Years later, RD was also discovered as a GA competitor for the Hsp90-interaction site by Schulte and co-workers in 1998 [16]. It was revealed that RD can mimic the ADP-bound conformation and interacts with Asp93 in a similar manner as that of GA. In the literature, RD was reported to have a greater affinity for ATP-binding pocket and adopts a different orientation when compared to GA [17]. It adopts a folded conformation with the macrocycle and aromatic ring is perpendicular instead of parallel geometric orientation [17]. However, in vivo, RD does not display anti-tumor activity and this is mainly due to its rapid metabolism and short half-life [18]. Therefore, several synthetic derivatives were created to find suitable alternatives that have the potential to maintain metabolic stability.

**Figure 3:**
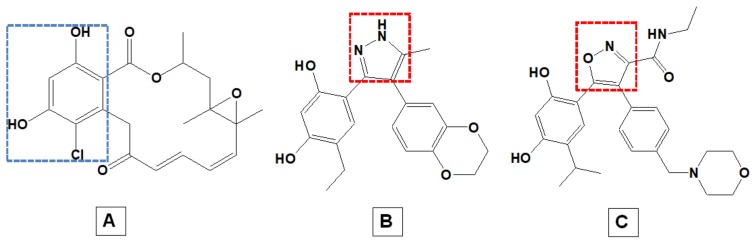
Structural representation of Hsp90 inhibitors: (**A**) RD, (**B**) CCT018159 and (**C**) NVP. Areas highlighted—blue: resorcinol ring and red: isoxazole scaffold.

The electrophilicity of RD was reduced by replacing 2’-ketone with oxime to increase the stability of the inhibitor [19]. It was observed that Hsp90 inhibition requires the resorcinol moiety of RD (Figure 3), as it behaves like the adenine ring of ATP [20]. Among 56,000 compounds screened by Workman and co-workers, CCT018159 was identified to contain a resorcinol-anchoring unit of RD [21]. Compound CCT018159 was subjected to further processing, which ultimately resulted in the generation of resorcinylic isoxazole amine NVP-AUY922/ VER52296 (NVP) [20].

Compounds possessing an isoxazole scaffold (Figure 3) have been nominated as potent Hsp90 inhibitors [22]. NVP compound is an isoxazole-based small molecule that is highly active and in literature, its suggested as a less toxic inhibitor. This compound inhibits Hsp90 by binding to the ATP-pocket. In doing so, ATP is unable to bind to Hsp90, normal physiological activity of the protein is impeded and it is no longer capable of binding to the target client protein [23,24]. Since its discovery, NVP has garnered a lot of attention as a potential Hsp90 inhibitor with 13 clinical trials underway, including nine phase II clinical trials [25,26]. Previous in vitro studies have shown that NVP is efficient against a variety of human derived cell lines and also inhibits progression of a wide range of tumors within in vivo models [20,23,24,27,28]. To date, no in silico studies have investigated the structural and dynamic characteristics underlying the inhibitory actions of NVP. 

Theoretical studies are important in expediting and saving resources when designing new inhibitors. Several in silico techniques have been used to accelerate drug discovery processes such as density functional theory (DFT), molecular docking and molecular dynamics (MD) simulation. These methods are used to predict the conceivable orientation of a ligand in the active site of a receptor as well as conformational changes experienced by the molecule during a given period of time [29]. The molecular mechanics/generalized born surface area (MM/GBSA) method is used to calculate the binding free energy of a ligand towards a protein of interest. The resultant data obtained from MM/GBSA can be used to provide in-depth perspectives into the binding parameters of a ligand to improve the development of lead inhibitors with enhanced pharmacodynamic profiles. This study investigated the interaction of RD and NVP within the ATP-binding site of Hsp90 *N*-terminal to provide an understanding of the structural and molecular dynamics of Hsp90 protease complexed with RD and NVP. We hope that the findings will facilitate future endeavors in the design and development of more potent Hsp90 inhibitors. 

## 2. Results and Discussion

### 2.1. DFT-Based Properties of RD and NVP

The results obtained from DFT calculations of the electronic properties of the two inhibitors are highlighted and relevant discussion provided hereafter.

#### 2.1.1. Geometric Analyses of the Compounds

**Figure 4:**
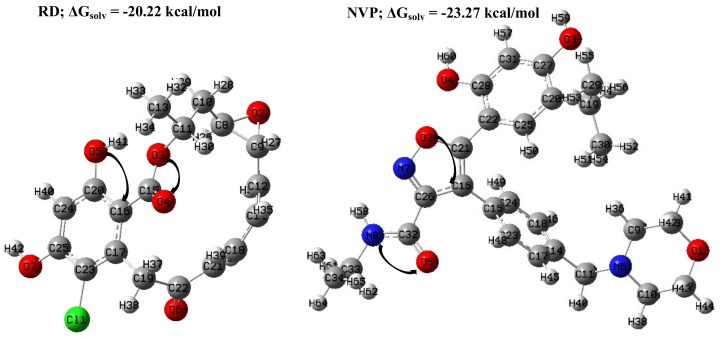
The optimized inhibitors at SMD/B3LYP/6-311G(d,p) level of theory indicating some charge transfer depicted through the NBO analysis. Atom color legend; green = chlorine, grey = carbon, white = hydrogen, blue = nitrogen and red = oxygen.

The application of B3LYP/6-311G(d,p) combination produced fully optimized geometries devoid of negative Eigen state value on examining their frequency in both vacuum and solvent phases. Solvation has been noted to contribute substantially to the general behavior of molecules [30] and this was considered using an implicit approach within the Gaussian package via SMD algorithm [31]. The energy change (ΔGsolv = ESMD – Egas) associated with solvent contribution could serve as an energetic measure [32]. Solvation free energy values of −20.22 and −23.27 kcal/mol were estimated for RD and NVP, respectively. Due to this significant difference, the parameters presented are output from solvent (water) phase calculations at SMD/B3LYP/6-311G(d,p) level of theory. Provided in Figure 4 are the fully optimized geometries of RD and NVP in implicit solvent showing their energy change.

A notable structural parameter often considered when analyzing the accuracy of optimized molecules is the bond length distance. The bond length distance of C–Cl is 1.765 Å, which is comparable to an approximate value of 1.76 Å from the literature [33]. Calculated interatomic C–C bonds are within 1.461 and 1.543 Å, which is comparable to experimental averaged value of 1.540 Å [34]. The benzene ring has C=C (partial double bond) distances of 1.382 to 1.435 Å in both inhibitors with an averaged value of 1.405 Å, which is in good agreement with the experimentally deduced value, 1.399 Å [34]. Calculated C=C (double bond) distances are within 1.346 and 1.375 Å, while C–O and C=O distances range from 1.353 to 1.447 Å and 1.215 to 1.235 Å, respectively; all these values are comparable to the experiment [34]. The measured interatomic distance of C–H was averaged and noted to be 1.088 Å, which is comparable to 1.090 Å from the literature [34]. The calculated interatomic distance of C–N ranges from 1.463 to 1.473 Å—these values are in order with ~1.470 Å for this distance in most study [33] while C=N partial and double bonds gave 1.344 and 1.313 Å, respectively. For N–O bond lengths, the values of 1.410 and 1.412 Å observed are comparable with 1.40 Å from literature, while O–H produced an average value of 0.969 Å, which is also close to experimental value of 0.960 Å [33]. The substantial correlation of the calculated interatomic distances with the experimental data reflects the accuracy of the selected theoretical level.

#### 2.1.2. Natural Bond Orbitals (NBOs) Analysis

Bond orbital analysis remains one of the most important parameters in the determination of electron density, charge transfer and atomic charge differences [35]. The orbital analysis was executed using the NBO program [36] implemented in the Gaussian 16 package. The NBOs represent the most effective way of presenting a picture of wavefunction ѱ, with the details of orbital being chosen mathematically, thereby allowing the greatest possible electron density of such compound [37,38].

**Figure 5:**
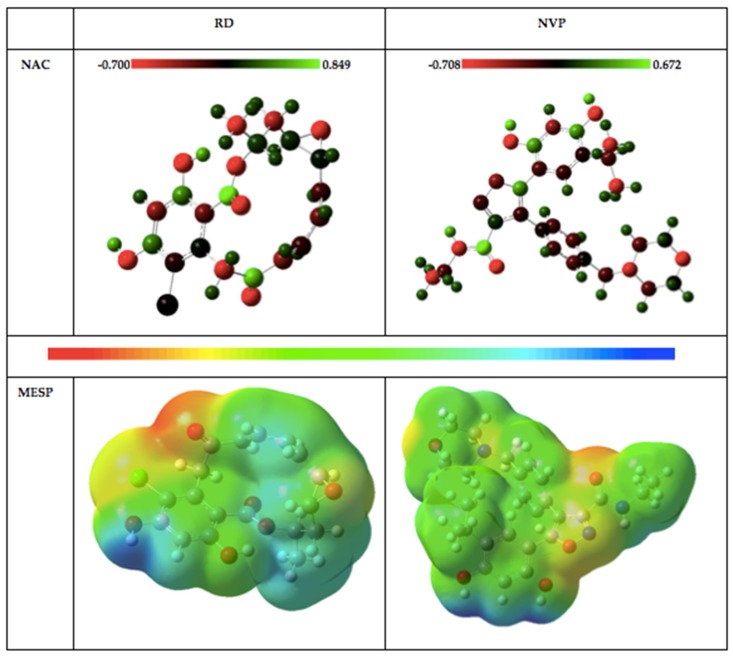
Natural atomic charge (NAC) distribution and molecular electrostatic potential (MESP) plots of the inhibitors at SMD/B3LYP/6-311G(d, p) DFT level.

Full population analysis was set up to estimate charge distribution within each inhibitor and the natural atomic charge (NAC) obtained through this calculation is presented in Table 1 for some selected atoms, while a pictorial representation of the charge distribution is provided in Figure 5. The chlorine, nitrogen and oxygen atoms are negatively charged while all the hydrogen atoms are positively charged. This feature is quite expected for these atoms. Many of the carbon atoms are negatively charged, including their methyl moiety. The six carbon atoms in the resorcinol ring of RD produced even charge (3 negative and 3 positive) distribution, while two carbon atoms from the isoxazole skeleton of NVP are positively charged.

Using 0.50 and 0.05 kcal/mol for printing and intermolecular threshold, respectively, the second-order perturbation theory analysis of Fock matrix [39] in NBO basis was calculated. The energy associated with this concept is known as the second order perturbation energy (E2), which is an important stability index. Investigating the nature of the electron delocalization and the energy involved could provide better insight on charge movement within the inhibitors [40].

Numerous possible interactions are usually obtained through the NBO calculation. In this calculation, the potential electron delocalization occurring from the interaction between the "filled" (donor) Lewis-type NBOs and the "empty" (acceptor) non-Lewis NBOs were noticed to have the highest values and thus considered. The E2 values associated with the lone pair (LP) of electron donor and anti-bonding (BD*) acceptor are provided in Table 2, along with the number of likely electrons available for delocalization in parentheses. Schematic representation of some possible electrons’ displacement, atom labels and symbols of the compounds are also indicated in Figure 4. It has been proposed that larger stabilization energy (E2) indicates greater interatomic charge transfer within a system [38,41]. 

Due to the similar nature of these two inhibitors, their stability energy values arising from (1, 2 or 3) LP donor displacement to (1 or 2) BD* acceptor are relatively high. However, it is quite fascinating to observe a unique electron movement from one lone pair of N8 electron to two anti-bonding of O5–C32 with the highest stability energy of 75.47 kcal/mol in NVP (Table 2). Recall that this inhibitor gave the highest solvation energy value (Figure 4).

#### 2.1.3. Molecular Electrostatic Potential (MESP) Plots

Atomic charge distributions on compounds play a crucial role in perturbing the electrostatic potentials of such compounds [42,43]. Plots from MESP surface analyses have been akin to reactivity and binding sites prediction [30,40,44,45,46]. The region of negative potential denotes the site for possible proton attraction or nucleophilic attack, while areas of positive potential are indications of prospective electrophilic addition at such regions. In this investigation, negative, positive and zero potential are displayed in colors red, blue and green, respectively. The MESP plots provided in Figure 5 reflect the availability of both nucleophilic and electrophilic regions. The former occurs majorly around the oxygen in C=O and C–O units, while the latter mainly occur on the oxygen in the OH group. It could be noticed that green color is well distributed around the compounds, thereby reflecting availability for weak electrophilic addition, while the yellow regions denote the distribution of a weak nucleophilic region. Analyses of the NBO and MESP surfaces of these inhibitors represent the availability of electrons for possible interaction with another group of atoms. 

#### 2.1.4. Analyses of the Non-Linear Optical Properties

Molecular polarizability (α) of a molecule reflects its global polarity which is inherent from unequal partial charges across its constituent atoms [47]. The hyperpolarizability (β) tensor of a compound is a function of electron delocalization resulting in anomalous non-linearity [48]. Polarizability and hyperpolarizability are nonlinear-optical (NLO) properties of a molecular system [49,50]. Displayed below Table 2 are the estimated values for the studied NLO properties of these inhibitors in which α and β are in electrostatic units (esu). α and β units are in atomic unit (a.u) from the Gaussian output and have been converted to electrostatic units (esu) (α: 1 a.u. = 0.1482 × 10^−24^ esu; β: 1 a.u. = 8.6393 × 10^−33^ esu).

The calculated α and βtot values are in favor of NVP having larger values than RD. The higher α value of 70.69 × 10^−24^ esu in NVP could be attributed to the notion on compounds with cyclic moiety (such as benzene), which tend to have larger α values [51]. The electric susceptibility of the compounds was calculated as a measure of the hyperpolarizability tensor. It was also noted that NVP produced the higher value of approximately 103.49 × 10^−29^ esu. A compound with higher β has been proposed to possess strong intramolecular charge transfer within its constituent atoms [52]. A higher β value has also been associated with the greater ability of such a system to polarize in response to an electric field, hence, its tendency to reduce the total electric field inside the system and consequently storing energy [53]. Hence, these NLO properties could serve as stability measures that reflect compound’s behavior in response to light.

Based on the conceptual properties investigated, it could be stated that NVP would be more reactive than RD when placed in the same biological molecule or group of atoms. In order to elucidate this hypothesis, MD simulations were carried out to investigate the binding interactions of both inhibitors with the *N*-terminal of Hsp90 protein.

### 2.2. Molecular Dynamics Simulations of Hsp90 with RD and NVP

Conformational changes experienced by Hsp90 are particularly crucial to the appropriate functioning of the protein and successive interactions with numerous other enzymes [54]. Therefore, studying the dynamic nature of Hsp90 by focusing on the internal atomistic motions can significantly contribute valuable information toward the scientific domain, in particular for chaperone proteins [18,20].

#### 2.2.1. Root of Mean Square Deviation (RMSD) 

**Figure 6:**
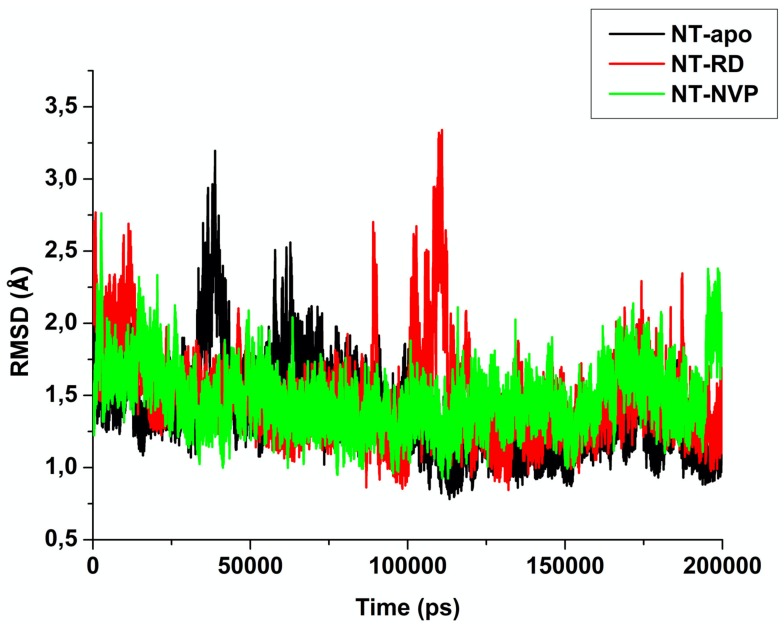
Root mean square deviation (RMSD) of free enzyme, radicicol (RD)-bound and NVP-bound Hsp90 recorded over 200,000 ps MD simulation.

Molecular dynamic simulations were performed for NT-apo, NT-RD and NT-NVP to establish the drug-binding pattern and to better understand the interactions of each inhibitor within the ATP-binding pocket. The mean root mean square deviation (RMSD) was used to determine the stability of studied protein–ligand complexes during the 200,000 ps MD simulation. The results obtained are graphically depicted in Figure 6. 

Throughout the simulation, the three systems showed acceptable stability. As observed in Figure 6, all three systems were stable throughout the 200,000 ps simulation with a small instability observed from NT-apo at approximately 40,000 and 60,000 ps. In the NT-RD system, instances of instability were detected at approximately 90,000 and 110,000 ps. Contrastingly, NT-NVP was stable from the beginning of the simulation and showed small degrees of instability at approximately 200,000 ps. The instabilities exhibited by the complex systems are a result of conformational changes that occur during the simulation. The Hsp90 protein has to have the ability to open and close during the ATP cycle for the decomposition and release of ADP. Hence, the apo protein has a higher degree of flexibility. Upon binding of RD and NVP, Hsp90 is not able to bind ATP due to the binding pocket being competitively occupied. Hence, the protein’s normal dynamical behavior is disrupted leading to restricted and rigid movements. This may ultimately lead to a loss of Hsp90 activity, thus rendering the protein non-functional.

#### 2.2.2. Root of Mean Square Fluctuation (RMSF)

**Figure 7:**
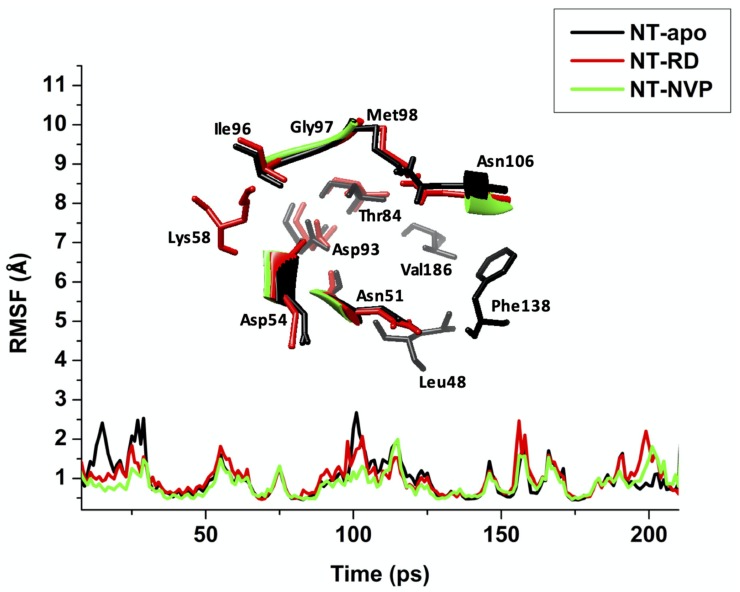
Average C-α fluctuations experienced by residues of the apo, RD and NVP bound conformation of Hsp90 throughout the 200,000 ps MD simulation.

The RMSF with regard to the averaged MD simulation conformation was utilized to signify the differences emerging in the flexibility of residues within a system. The RMSF of all residues were calculated for backbone structure flexibility and is represented in Figure 7. 

The RMSF was calculated from MD trajectories to provide an insight into the residue flexibility of various regions of three systems within 200,000 ps (Figure 7). A similar trend in residue fluctuations was observed for all three simulations. Overall, both the bound conformations showed less RMSF fluctuations compared to the apo (NT-apo; 1.17, NT-RD; 1.13 Å and NT-NVP; 1.06 Å). These findings indicate that the backbone of the bound conformations is more stable than that of the free enzyme. Residues 48–58, 96–106, 148–170 and 184–186, which are contributing to a pocket, have shown less degree of fluctuation for NT-NVP, which suggests higher affinity compared to NT-RD. As evident from RMSD and RMSF, NT-NVP is the most stable complex when compared to the NT-RD complex, this could be related with the DFT study in which NVP is more stable than RD.

#### 2.2.3. Radius of Gyration (RoG)

**Figure 8:**
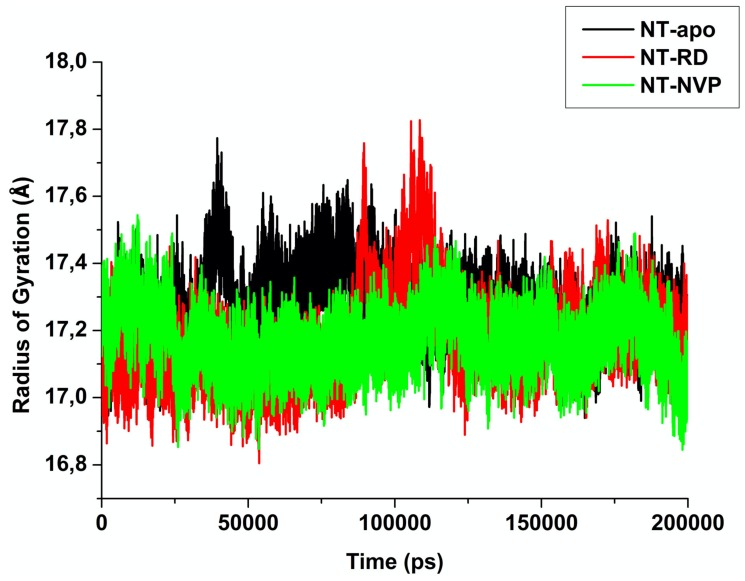
The RoG of NT-apo, NT-RD and NT-NVP recorded over 200000 ps MD simulation.

The RoG assesses the compactness of a protein structure while simultaneously providing insight into the stability of the studied complexes. The comparative RoG for NT-apo, NT-RD and NT-NVP is shown in Figure 8, the average values for the NT-apo, NT-RD and NT-NVP were 17.32 ± 0.101 Å, 17.22 ± 0.132 Å and 17.20 ± 0.0969 Å, respectively. The results obtained indicate that upon the binding of NVP to the *N*-terminal of Hsp90, the protein conformation was constant throughout the simulation, while NT-RD showed a conformational alteration at around 100,000 ps. It is observed that the RoG values for the NT-apo and NT-bound systems are fairly similar, there only being a 0.12 Å change, ultimately forming a relatively stable enzyme. Hence, this justifies substantial biomolecular flexibility of the NT-apo and NT-RD in comparison to NT-NVP. This strongly indicates that Hsp90 ATP-binding pocket possesses rigid structural stability when bound to an inhibitor at the calibre of NVP. In the literature, RD has been shown to inhibit Hsp90 (IC_50_ < 1μM), while NVP inhibits Hsp90 (IC_50_ < 0.1μM) [55,56]. This also warrants the overall stability of NT-NVP.

#### 2.2.4. Principal Component Analysis (PCA)

**Figure 9:**
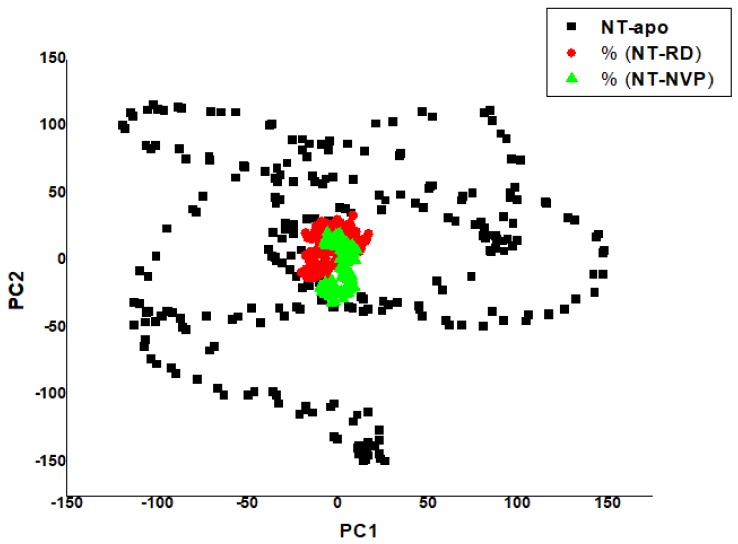
Projection of PC1 over PC2 for NT-apo, NT-RD and NT-NVP conformations.

Figure 9 shows a PCA scatter plot generated for the NT-apo and NT-bound complexes showing a significant difference between apo and bound systems, as evident from the characteristic structures plotted along the direction of two principal components. The NT-apo occupies a larger phase space and exhibits a higher fluctuation compared to the bound complexes. Among the two bound systems (NT-RD and NT-NVP), NT-NVP showed a slightly lesser fluctuation compared to NT-RD, due to NVP having a higher affinity with an active site compared to RD. This results in NT-NVP being more compact compared to NT-RD, which also agrees with RMSD, RMSF and RoG.

#### 2.2.5. MM/GBSA Binding Free Energy Calculation

The MM/GBSA method was used to calculate the overall binding free energies for both NT-RD and NT-NVP complexes over the 200,000 ps MD trajectory, as shown in Table 3.

Table 3 present the binding free energy *(∆G_bind_)* of NT-RD and NT-NVP, which was calculated to be −28.9 kcal/mol and −49.4 kcal/mol, respectively. The difference in binding free energy (−20.5 kcal/mol) between the NT-RD and NT-NVP is quite significant. Interactive forces such as electrostatic [(−45.6 kcal/mol) and van der Waals (−52.5 kcal/mol)] in NT-NVP and electrostatic [(−31.9 kcal/mol) and van der Waals (−29.7)] NT-RD highly contributes toward the total binding energies of each system. The favorability of NT-NVP suggests that NVP has a stronger interaction with ATP-binding pocket amino acid residues when compared to RD. The findings generated from this method further support NVP’s potential as a Hsp90 inhibitor [10,20,25]. The IC50 is not a direct indicator of affinity, although the indirectly related to confirm that NVP has more potency towards Hsp90 compared to RD. Entropy effects play an important role in drug–target interactions, but the entropic contribution to ligand-binding affinity is often omitted by endpoint binding free energy calculation methods such as MM/GBSA and MM/PBSA due to the high computational expense of normal mode analysis (NMA) [57,58]. The binding free energies estimated by including the truncated-NMA entropies based on the MD trajectories have been reported to give the lowest average absolute deviations against the experimental data among all the tested strategies for both MM/GBSA and MM/PBS [57,58]. There have been no reports on deviations against binding free energies estimated without entropy calculations. Therefore, binding free energy estimations are reported without entropy calculations.

**Figure 10:**
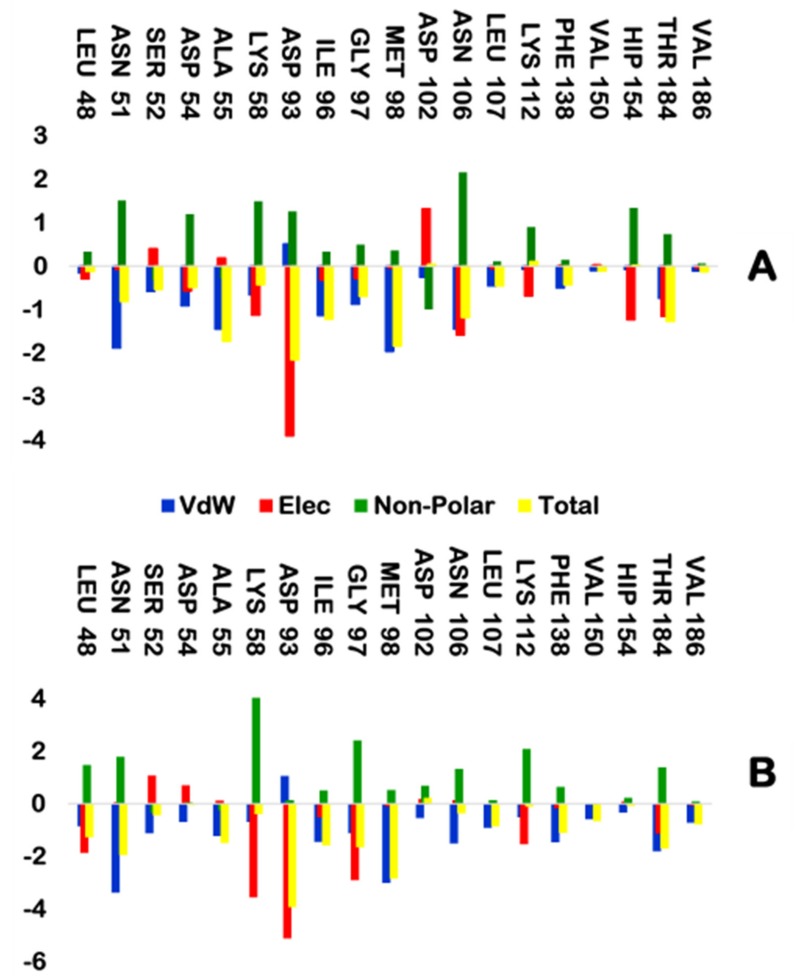
The per-residue free energy decomposition of (**A**) NT-RD and (**B**) NT-NVP.

The binding free energy was decomposed into the unit contributions of each active site residue of NT-RD and the NT-NVP complexes, as represented graphically in Figure 10. The residues contributing the most to the NT-RD complex include Asp 93 [−3.9 kcal/mol (elec)], Asn 51 [−1.9 kcal/mol (vdw)], Ala 55 [−1.5 kcal/mol (vdw)], Lys 58 [−1.1 kcal/mol (elec)], Ile 96 [−1.1 kcal/mol (vdw)], Met 98 [−2.0 kcal/mol (vdw)], Gly 97 [−0.9 kcal/mol (vdw)] Asn 51 [−1.5 kcal/mol (vdw)], [−1.6 kcal/mol (elec)] and Thr 184 [−1.2 kcal/mol (elec)]. The residues that contribute the most energy in the NT-NVP complex include Asp 93 [−5.1 kcal/mol (elec)], Leu 48 [−0.9 (vdw)], [−1.866 kcal/mol (elec)] Asn 51 [−3.4 kcal/mol (vdw)], Ala 55 [−1.2 kcal/mol (vdw)], Lys 58 [−3.6 kcal/mol (elec)], Ile 96 [−1.4 kcal/mol (vdw)], Met 98 [−3.0 kcal/mol (vdw)], Gly 97 [−1.1 kcal/mol (vdw)], [−2.9 kcal/mol (elec)], Asn 106 [−0.1.5 kcal/mol (vdw)], Lys 112 [−1.5 kcal/mol (elec)], Phe 138 [−1.5 kcal/mol (vdw) and Thr 184 [−1.8 kcal/mol (vdw)], [−1.1 kcal/mol (elec)]. These findings further indicate the NT-NVP binding free energy being favorable over NT-RD complex. Furthermore, Asp 93, the prominent elec contributor observed to project a greater impact on the total binding energy compared to other residues followed by Gly 97. These residues are regarded as key components of the ATP-binding pocket [29,59].

**Figure 11:**
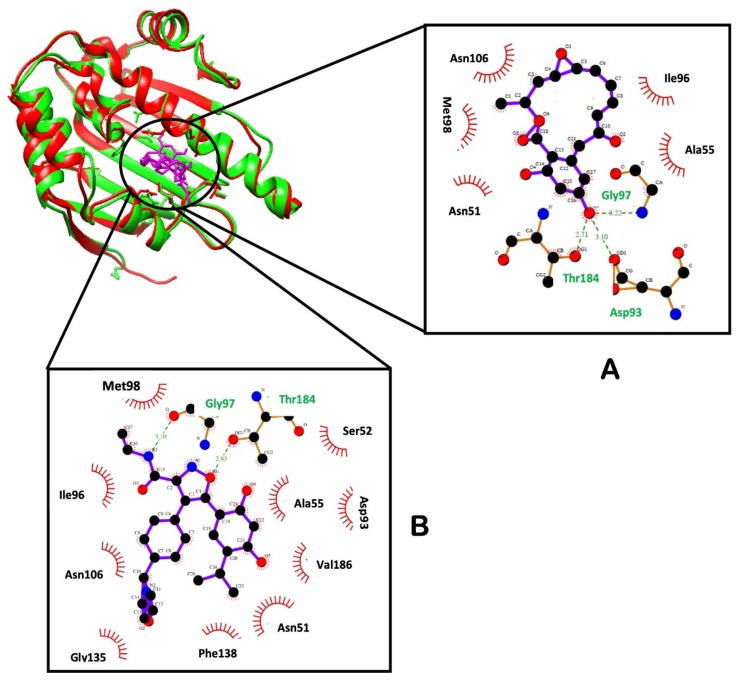
The interactions of (**A**) RD and (**B**) NVP with Hsp90 residues within the ATP-binding pocket (plotted by LigPlot).

Illustrated in Figure 11 are the interactions of RD and NVP with the active residues of NT Hsp90 protein. The nature of the enzyme-ligand interaction could offer a better understanding of the binding landscape of a ligand to a target. It was generally noticed that Gly 97 and Thr 184 from the ATP-binding pocket of NT Hsp90 form hydrogen bonds with both RD and NVP.

As shown in Figure 11, both ligands interacted with similar amino acids within the ATP-binding site. The binding site consists of a hydrophobic pocket and a hydrogen bond receptor region, which was predicted from the MESP analysis of the inhibitors (Figure 5). Due to the presence of acidic residues, this specific region maintains a negative charge. Hydrogen bond donor groups of the ligands interact with this region, thus essentially facilitating ligand binding to the ATP-binding site of Hsp90 [60]. The active site also encompasses hydrophobic residues, and the ligand molecules actively interact with these residues by means of van der Waals interactions. Hydrogen bonds are formed between NVP and two residues—Gly 97 and Thr 184—and ten residues forming van der Waals interactions. Meanwhile, RD showed hydrogen bond formation with Gly 97, Asp 93 and Thr 184, with five residues forming van der Waals interactions. Cumulatively, NT-NVP is suggested as the favorable ligand due to a greater binding affinity and increased stability, as rendered by results obtained from RMDS, RoG and RMSF.

#### 2.2.6. Hydrogen Bond Network Profile

Hydrogen bonds (H-bonds) are ubiquitous in nature. They play a central role in biological systems and in maintaining the structural integrity of proteins [61]; protein ligand interaction and catalysis [61]. To further investigate the impact of RD and NVP binding on Hsp90 *N*-terminal, the evolution of hydrogen bond distances and fraction were monitored between amino acid residues interacting with RD and NVP in the active site for 200-ns simulations (Table 4).

The primary residues constituted hydrogen bonds; Asp 93, Gly 97, and Thr 184. Such amino acids were identified as key residues for binding site Hsp90 *N*-terminal [29,59]. The greater the interaction between the ligand and these main residues therefore plays an important role in a ligand’s potency. The findings in Table 4 agree with LigPlot in Figure 11, showing the atoms responsible for the hydrogen interactions with more information on which atom is an H-donor and H-acceptor. Looking closely at Asp93-NVP interaction, it was found to have a 99.6 percent fraction whereas Asp93-RD is 59.6 percent interaction, which correspond to the energy distribution (Figure 10), with the interactions between Asp93-NVP and Asp93-RD being −5.1 and −3.9 kcal/mol (elec), respectively. The percentages of Gly97-NVP and Gly97-RD were 32.4% and 14.6%, respectively (Table 4), while the energy distribution was −1.1 and −0.9 kcal/mol (elec), respectively for Gly97-NVP and Gly97-RD. The fractions for Thr184-NVP and Thr184-RD were 31.3% and 27.1%, respectively, which also supports the −1.1 and −1.2 kcal / mol (elec) energy distribution for Thr184-NVP and Thr184-RD. These results indicate that the NT-NVP bind free energy complex and that stability is greater than that of the NT-RD complex.

## 3. Computational Methodology

### 3.1. DFT Calculations

All calculations were executed within the Gaussian 16 Rev. B01 (G16) program package [62] at 298.15K in vacuum and solvent (water). Becke 3 plus Lee, Yang, Parr (B3LYP) [63,64] hybrid exchange DFT algorithm was used in combination with 6-311G(d,p) [65] basis set. The selected level of theory is sufficient for main group molecular properties evaluation [66]. The compounds were modeled with GaussView 6.0.16 [67] and their geometries were fully optimized at B3LYP/6-311G (d,p) level of theory. Vibrational frequency calculation was done to ensure that the structures are truly minima with no negative Eigenvalue.

#### 3.1.1. Charge Distribution and Electrostatic Potential

The second-order Fock matrix was computed to assess the donor−acceptor interaction and compute charge distribution arising from the natural bond orbital (NBO) [39] calculation. The NBO analysis provides a unique way of representing delocalization of electron, distribution of atomic charges and interatomic interaction of atoms within a compound [39]. In addition, the energy associated with the transfer of charges within an inhibitor could be obtained from the NBO analysis. This energy is called the second-order perturbation energy (E^2^) and it is an important stability index. In order to estimate the total electrostatic effect inherent from charge distribution on each atom within the inhibitors, molecular electrostatic potential (MESP) surface was generated using the Merz–Kollman approach [68] and the MESP plot was generated with GaussView.

#### 3.1.2. Non-Linear Optical Properties

Polarizability (α) [50] and the first static hyperpolarizability (β) [49], which are non-linear optical (NLO) properties, were also calculated. This is applied to investigate the optical performance of these compounds under visible light. Using Equation (1), the magnitude of the total first static hyperpolarizability was calculated from the Gaussian 16 output in 3 × 3 × 3 matrices. These parameters were obtained through the frequency calculation at B3LYP/6-311G (d,p) level of theory.

(1) $$\beta_{\mathrm{tot}}={\left[{\left(\beta_{\mathrm{xxx}}+{\;\beta }_{\mathrm{xyy}}+{\;\beta }_{\mathrm{xzz}}\right)}^{2}+{\left(\beta_{\mathrm{yyy}}+{\;\beta }_{\mathrm{yzz}}+{\;\beta }_{\mathrm{yxx}}\right)}^{2}+{\left(\beta_{\mathrm{zzz}}+{\;\beta }_{\mathrm{zxx}}+{\;\beta }_{\mathrm{zyy}}\right)}^{2}\right]}^{\frac{1}{2}}$$

### 3.2. Molecular Dynamics Simulations Protocols

#### 3.2.1. System Operations and Molecular Docking

Systems operations were employed in molecular docking, prediction of ligand interactions and in the assessment of binding free energies of apo Hsp90, NT-RD and NT-NVP using AutoDock Tools software. The X-ray crystal structure of Hsp90 protein complexed with ATP was obtained from RCSB Protein Bank (PDB ID: 3T0Z) [69]. The complex was thereafter separated using UCFS Chimera to prepare the Hsp90 protein for docking. Both RD and NVP were individually docked to Hsp90 ATP-binding site using AutoDock Tools and AutoDock vina [70,71]. Non-polar hydrogens were merged after calculating Kollman atom charges and their files were saved as pdbqt [70]. The ligands were also prepared and saved as pdbqt. Water molecule parameters were added to AD4-bound and AD4-parameter files before calculating the grid maps by AutoGrid 4.2. Missing residues were added using UCSF Chimera [72]. Avogadro software was also used in this study to prepare the ligands and Hsp90 protein for MD simulation [73].

#### 3.2.2. System Preparation

An Amber 14 software package that runs on a graphic progressing unit (GPU) was used to setup the simulation [74,75,76]. The receptor and ligands were optimized using the Amber 14 modules ANTECHAMBER and LEAP [77,78,79]. The FF14SB force field on Amber 14 was used to parameterize the protein system [80,81]. Each system is enclosed in the TIP3P water box with the protein atoms located 10 Å between the protein surface and the box boundary within the period of simulations [81]. The minimizations, heating and equilibration steps carried out in this study is extensively explained in several published papers [82,83,84]. All the trajectories were then saved every 1 ps and thereafter analyzed. The CPPTRAJ and PTRAJ modules [85,86] were used for analysis: root mean square deviation (RMSD), root mean square fluctuation (RMSF) and Radius of Gyration (RoG). The graphs were plotted using the Origin data analysis tool [86] while visualizations were done in UCFS Chimera [72].

#### 3.2.3. Root Mean Standard Deviation

A root mean square deviation (RMSD) was used in the analysis of macromolecular structures and dynamics within a given MD simulation [87]. The RMSD is computed using Equation (2).

(2) $$RMSD=\sqrt{\frac{\sum_{i=1}^{N}d_{i}^{2}}{N}\;}$$

where *di* is the distance between atom *i* and the mean position of the N equivalent atoms. This calculation was performed for the 3D backbone heavy atoms of the protein structure.

#### 3.2.4. Root Mean Standard Fluctuation

A root mean standard fluctuation (RMSF) is the fluctuation of individual enzyme residues with respect to their average position within a measured MD trajectory [88]. The RMSF provides insight into residue dynamic behavior of various regions of the apo and Hsp90 complexed with RD and NVP. The RMSF analysis was mathematically calculated as stated in Equation (3).

(3) $${\mathrm{sRMSF}}_{i}=\frac{\left({\mathrm{RMSF}}_{i}-\mathrm{RMSF}\right)}{\sigma \left(\mathrm{RMSF}\right)}$$

where RMSF_i_ represents the RMSF of the i^th^ residue, from which the average RMSF is subtracted. This is then divided by the RMSF’s standard deviation [σ(RMSF)] to yield the resultant standardized RMSF (sRMSF_i_).

#### 3.2.5. Radius of Gyration

The compactness of a protein within a given MD simulation is referred to as RoG, which is a description of the RMSD of atoms from the common center of gravity of a given enzyme molecule. The RoG is determined using Equation (4).

(4) $$r^{2}g=\frac{\sum_{i=0}^{n}w_{i}{\left(r_{i}-{\;r}^{-}\right)}^{2}}{\sum_{i=1}^{n}w_{i}}$$

where r_i_ is the position of the ith atom and r the center mass atom i. The number of frames in each trajectory gives the RoG values, which are used to calculate the mean value.

#### 3.2.6. Binding Energy Calculations

The molecular mechanics/generalized born surface area (MM/GBSA) approach, developed from Amber 14 software package that runs on a GPU, was used to compute the binding free-energy profiles of RD and NVP bound to Hsp90 protease [89,90,91]. This form of analysis provides valuable insight into the association of the protein-ligand of a complex and is considered to be the end-point energy calculation. In this study, the binding free energy was averaged over 1000 snapshots, which were extracted from the entire 200,000 ps trajectory. The MM/GBSA computes the binding free energy based on the equations below:

(5) $$\Delta G_{\mathrm{bind}}=G_{\mathrm{complex}}-G_{\mathrm{receptor}}-G_{\mathrm{ligand}}$$

(6) $$\Delta G_{\mathrm{bind}}=E_{\mathrm{gas}}+G_{\mathrm{sol}}-\mathrm{TS}\;$$

(7) $$E_{\mathrm{gas}}=E_{\mathrm{int}}+E_{\mathrm{vdw}}+E_{\mathrm{ele}}$$

(8) $$G_{\mathrm{sol}}=G_{\mathrm{GB}}+G_{\mathrm{SA}}$$

(9) $$G_{\mathrm{SA}}=\gamma \mathrm{SASA}$$

where E_gas_ denotes gas-phase energy, evaluated directly from the FF14SB force terms. The *E_MM_* and *E_int_* signify the molecular mechanic energy and internal energy, respectively. The *E_ele_* and *E_vdw_* indicate the electrostatic and van der Waals contributions, respectively. *G_sol_* denotes solvation free energy, and can be decomposed into polar and nonpolar contribution states. *G_GB_* denotes polar solvation contribution, determined by solving the GB equation, whereas G_SA_, the nonpolar solvation contribution, is estimated from the solvent accessible surface area (SASA), determined using a water probe radius of 1.4 Å. *T* and *S* correspond to temperature and total solute entropy, respectively. The solute and solvent dielectric constants are set to 1 and 80, respectively [89]. The MM/GBSA method was used, analysing the interaction energy for each residue, to determine the contribution of each of the amino acids towards total binding free energy profile between RD or NVP and Hsp90.

## 4. Conclusions

Cancer is a continuously escalating global burden. Current anti-cancer treatments are often deemed ineffective in eradicating cancerous cells and frequently possess undesirable adverse effects that negatively impact the health of ailing patients. The Hsp90 protein is established as a fundamentally important contributor in malignant development and progression. For this reason, Hsp90 is regarded as an attractive target in anti-cancer research. In the literature, RD and NVP are known Hsp90 inhibitors; however, there is a lack of knowledge surrounding the interactions of these compounds with Hsp90, from an atomistic perspective. The selected inhibitors were initially investigated using the DFT algorithm. Their physical features were examined in the form of interatomic bond distance analyses and energetics in a solvent. Calculated bond length distances agreed with the experimental data, while bulk solvent contribution was higher in NVP compared to RD. The impact of charge transfer and distribution within the inhibitors provided more insight on their stability. The important charge transfer arises from the lone pair of electrons to the anti-bonding orbital within the inhibitors, in which the highest value was observed in NVP. MESP plots gave a clearer picture of the nature of the inhibitors and act as a binding site prediction parameter. The calculated NLO parameters proved to be substantial in evaluating the optical properties of the compounds and NVP gave favorable outcome than RD.

Through in silico MD simulation, the structural and dynamical behaviors of RD and NVP within the Hsp90 ATP-binding pocket was investigated. As evident from RMSD, RMSF and RoG profile, a higher system stability, a low degree of residue flexibility and constant compactness for NT-NVP in comparison to NT-RD. The NT-NVP showed a less fluctuation degree in the region 93–106 and 184–186. Within these regions are Asp93, Gly97 and Thr184 residues, which were observed in LigPlot to form hydrogen for NT-RD, while Gly97 and Thr 184 also formed hydrogen bonds for NT-NVP. The per-residue free energy contribution diagram showed that Asp93, Gly97 and Thr184 have high electrostatic interactions on NT-NVP compared to NT-RD, which contributes to the significant affinity and stability of NT-NVP. The NT-NVP binding free energy was shown to be smaller than that of NT-RD. Therefore, the MM/GBSA and MD simulations agree that NVP is a stronger binding inhibitor than RD, with entrusted stability within the ATP-binding site of Hsp90 *N*-terminal. Therefore, NVP can be considered as a good candidate for Hsp90 inhibition. The findings of this study provide a platform that will allow targeted design and development of more potently effective Hsp90 inhibitors for anti-cancer therapy. 

## Figures and Tables

**Table 1:** Natural atomic charge (in esu) distribution on some selected atoms within the optimized inhibitors at SMD/B3LYP/6-311G(d,p) level of theory.

Atom	RD	NVP
Cl1	−0.019	O1, −0.618
O2	−0.584	−0.320
O3	−0.610	−0.704
O4	−0.659	−0.701
O5	−0.700	−0.708
O6	−0.644	N6, −0.533
O7	−0.679	N7, −0.176
C15	0.849	N8, −0.587

Atoms’ labels and symbols are presented in Figure 4 and esu = electrostatic unit

**Table 2:** The second-order perturbation energies E^2^ (kcal/mol) corresponding to the important charge transfer interaction (donor → acceptor) within the inhibitors and their NLO properties at SMD/B3LYP/6-311G(d,p) level of theory.

RD			NVP		
Donor	Acceptor	E^2^ (kcal/mol)	Donor	Acceptor	E^2^ (kcal/mol)
LP (3) Cl1	BD* (2) C17–C23	10.97	LP (2) O2	BD* (2) N7–C26	16.56
LP (2) O2	BD* (1) C9–H27	5.46	LP (2) O2	BD* (2) C16–C21	30.08
LP (2) O3	BD* (2) O4–C15	40.86	LP (2) O3	BD* (2) C27–C31	29.49
LP (2) O4	BD* (1) O3–C15	31.43	LP (2) O4	BD* (2) C22–C28	29.35
LP (2) O4	BD* (1) C15–C16	16.40	LP (2) O5	BD* (1) N8–C32	20.69
LP (2) O5	BD* (2) C16–C20	35.57	LP (2) O5	BD* (1) C26–C32	18.24
LP (2) O6	BD* (1) C19–C22	20.31	LP (1) N6	BD* (1) C10–H35	6.97
LP (2) O6	BD* (1) C21–22	16.89	LP (1) N6	BD* (1) C11–H39	6.97
LP (2) O7	BD* (2) C24–C25	32.79	LP (1) N8	BD* (2) O5–C32	75.47
α (×10^−24^ esu)	β_tot_ (×10^−29^ esu)		α (×10^−24^ esu)	β_tot_ (×10^−29^ esu)	
51.857	18.333		70.692	103.494	

Atom label and symbol are presented in Figure 4.

**Table 3:** Summary of molecular mechanics/generalized born surface area (MM/GBSA)-based binding free energy contributions and IC_50_ of RD and NVP bound to the ATP-binding pocket of Hsp90.

	Energy Components (kcal/mol)					Expt (μM)
	ΔEvdw	ΔEelec	ΔGgas	ΔGsolv	ΔGbind	IC50
NT-RD	−31.9 ± 3.3	−29.7 ± 7.2	37.0 ± 5.7	4.4 ± 0.3	−28.9 ± 4.5	1
NT-NVP	−52.5 ± 4.1	−45.6 ± 7.2	55.3 ± 6.1	−6.5 ± 0.6	−49.4 ± 3.9	0.1

*ΔE_elec_*: electrostatic, *ΔE_vdw_*: van der Waals, *ΔG_bind_*: calculated total free binding energy, *ΔG_gas_*: gas phase interaction, and *ΔG_solv_*: solvation free energy. *Expt*: Experimental inhibition affinity from literature [55,56].

**Table 4:** Percentage (%) Occupancy and average distance (Å) between the ligand and prominent active site residues calculated over simulation time.

	#Acceptor	DonorH	Donor	Frames	Percentage (100 %)	Average Distance
**NT-RD**	ASP 93@OD1	RD@H1	RD@O3	119247	59.6	2.6309
	THR 184@OG1	RD@H1	RD@O3	54267	27.1	2.7190
	RD@O3	GLY 97@H	GLY 97@N	29273	14.6	2.9167
**NT-NVP**	ASP 93@OD2	NVP@H23	NVP@O4	199273	99.6	2.6117
	GLY 97@O	NVP@H15	NVP@N3	64888	32.4	2.9001
	NVP@O1	THR 184@HG1	THR 184@OG1	62686	31.3	2.8657
